# The homology gene *BtDnmt1* is Essential for Temperature Tolerance in Invasive *Bemisia tabaci* Mediterranean Cryptic Species

**DOI:** 10.1038/s41598-017-03373-w

**Published:** 2017-06-08

**Authors:** Tian-Mei Dai, Zhi-Chuang Lü, Wan-Xue Liu, Fang-Hao Wan, Xiao-Yue Hong

**Affiliations:** 1 State Key Laboratory for Biology of Plant Diseases and Insect Pests, Institute of Plant Protection, 100193, Beijing, 100193 P.R. China; 2 Center for Management of Invasive Alien Species, Ministry of Agriculture, Beijing, 100193 China; 3 Department of Entomology, College of Plant Protection, Nanjing Agricultural University, Nanjing, Jiangsu 210095 P.R. China

## Abstract

The *Bemisia tabaci* Mediterranean (MED) cryptic species has been rapidly invading most parts of the world owing to its strong ecological adaptability, particularly its strong resistance to temperature stress. Epigenetic mechanisms play important roles in mediating ecological plasticity. In particular, DNA methylation has been the focus of attempts to understand the mechanism of phenotypic plasticity. The relationship between temperature and DNA methylation and how it affects the adaptability of invasive insects remain unknown. To investigate the temperature resistance role of DNA methyltransferase 1 (Dnmt1) in MED, we cloned and sequenced *BtDnmt1* homology and identified its functions under various temperature conditions. The full-length cDNA of MED *BtDnmt1* homology was 5,958 bp and has a 4,287 bp open reading frame that encodes a 1,428-amino-acid protein. *BtDnmt1* mRNA expression levels were significantly down-regulated after feeding with dsRNA. Furthermore, after feeding with ds*BtDnmt1*, the MED adults exhibited significantly higher mortality under temperature stress conditions than the controls, suggesting that MED BtDnmt1 homology plays an essential role in the temperature tolerance capacity of MED. Our data improve our understanding of the temperature resistance and temperature adaptability mechanisms that have allowed the successful invasion and colonization of various environments by this alien species.

## Introduction

Epigenetics is a factor that changes the phenotype of an organism without changing its DNA sequences. Epigenetic modifications can cause heritable variations in ecology, such as productivity and stability, phenotypic plasticity and habitat differentiation^1–3^. Moreover, phenotypes caused by epigenetics are reversible alterations that mediate the rapid plastic responses of organisms to environmental perturbations and can increase the capacity of organisms to adapt to environmental stresses^4–6^. Therefore, epigenetics has gained unprecedented interest in recent years, not only as a subject of basic ecological research but also representing an overlooked level of rapid adaptation that must be incorporated into adaptable species, particularly exotic species. Many emerging invasive species display evidence of rapid adaptation in invaded environments and flourish even with low levels of sequence-based genetic variation^7–9^.

Epigenetics provides one of rational mechanisms for the link between phenotype and genotype^10–12^ and is accomplished by DNA methylation, histone modifications, chromatin remodelling, and non-coding RNA machinery. Epigenetics is an important mechanism that results from a fast and flexible system that is sensitive to environmental stress and mediates heritable and reversible changes in gene expression patterns. Important advances include a study on the effects of temperature on the epigenome and gene expression in the *Caenorhabditis elegans* germline, which was closely monitored by small RNA pathways^13^. A correlation between changes in DNA methylation levels and cold stress tolerance was reported in the cores of nucleosomes in maize root tissues after exposure to various environmental cues^14^. Of note, DNA methylation, one of the most important epigenetic modifications, might occur rapidly in response to large-scale temperature changes and, thus could represent a potential method of coping with temperature stress over short time scales. In invertebrates, DNA is methylated at cytosine residues to form 5-methylcytosine by several evolutionarily conserved enzymes, called DNA methyltransferases (Dnmts). Dnmt families have been further divided into three classes— Dnmt1, Dnmt2, and Dnmt3—based on the nature of their activity^15^. Dnmt2 was originally misclassified and is now deemed to be implicated in transfer RNA methylation^16, 17^. Dnmt3 is involved in de novo methylation and establishes new methylation patterns during gametogenesis; it consists of three genes in vertebrates: *Dnmt3a*, *Dnmt3b* and *Dnmt3L* ^18, 19^. Dnmt1 has a 5- to 30-fold preference for hemimethylated DNA substrates over unmethylated substrates and has been implicated in the maintenance of previously established methylation patterns across cell generations^20, 21^. Dnmt1 contains three isoforms, Dnmt1o, Dnmt1s and Dnmt1p^22, 23^.

The primary function of Dnmt1 in vertebrates is to repair DNA methylation, and these enzymes can copy DNA methylation patterns from the parental DNA strand to the newly synthesized daughter strand^24^. In mammals, gene-specific DNA methylation patterns could be altered under conditions of environmental stresses. Studies suggest that rats’ offspring fed in the presence of a chronic constriction injury or by adult prenatally stressed mother rats showed significant increases in behavioural abnormalities concurrent with increased *Dnmt1* expression^25, 26^. Moreover, studies in humans^27^, rats^28^ and pigeons^29^ after exposure to arsenic trioxide, plumbum and avermectin, respectively, have revealed that poison induced hypomethylation of DNA is accompanied by decreased *Dnmt1* levels.

Skjærven *et al*.^30^ found that *Dnmt1* in Atlantic cod was sensitive to acute thermal stress and displayed significantly lower expression levels but was not affected by continuous thermal stress. Interestingly, thermal stress caused lower methylation just prior to hatching. Furthermore, *Dnmt1* also expresses de novo methylation activity. In insects, both *Schistocerca gregaria* ^31^ and *Bombyx mori* ^32^ contained the *Dnmt1* gene without the *Dnmt3* gene, indicating that *Dnmt1* might play roles in maintaining methylation and de novo methylation. Furthermore, widespread evidence suggests that *Dnmt1* might provide vital contributions to developmental and phenotypic variations^33, 34^. However, few studies have been conducted in insects due to the lower percentage of methylated cytosines in insects (0–10%) than in mammals (3–10%) and plants (up to 50%)^35, 36^. To date, studies on *Dnmt1* have mostly focused on the model insect *Drosophila melanogaster* and on social insects^37^. The roles of *Dnmt1* in environmental stress are important, but experimental evidence in other insects, such as invasive insects, is scarce.

Insects, which are ectothermic species, are sensitive to environmental temperatures and have a limited capability for thermoregulation. Their geographical distribution and dispersion are largely dependent on environment temperatures. Global warming is expected to accelerate insect migration and may also provide more favourable environments for invading insects^38^. Studies have shown that successful invasion mechanisms by insects include their strong physiological and ecological tolerability and plasticity and a rapid adaptive response to environmental changes^39, 40^. Huang *et al*.^41^ reported that the speed of invasion was positively correlated with successful invasion and had significant effects on insect invasion, through changing their basic characteristics and life cycles to increase their temperature adaptability and plasticity. Furthermore, recent genetic studies have found that rapid evolutionary adaptation to novel environments involve responses to climate changes might occur within several generations^42^, indicating that climate change could facilitates the establishment of invasive species. Comparisons between the epigenetic variabilities of native and invasive insects could show marked asymmetry in the distribution of variability, with epigenetic depauperacy evident during invasion^43^. Studying epigenetic complexes during rapid adaptation events would allow us to gain greater insight into understanding the molecular mechanisms linking genotypes and environmental stresses in invasive species.

The whitefly *Bemisia tabaci* (Gennadius) (Hemiptera: Aleyrodidae) is a cosmopolitan, polyphagous invasive pest that causes damage to many crops through direct feeding, depositing honeydew and transmitting plant viruses^44, 45^. *B. tabaci* is a species complex consisting of many morphologically indistinguishable but reproductively isolated cryptic species, comprising at least 36 morphologically indistinguishable species, including the Mediterranean cryptic species (MED) and the Middle East-Asia Minor1 cryptic species (MEAM1)^46–48^. MED, which was first detected in China in 2003^49^, invaded many provinces over the next 10 years and gradually became widespread, displacing local cryptic species (such as AsiaII3) and MEAM1, especially in the northern part of the country^50^. As an invasive species, MED has immense vast potential to adapt to a wide range of environmental temperatures^51–53^, which allows it to successfully colonize and disperse after invasion and to occupy habitats in a wide latitude range. Previous studies^54^ have found that MED can significantly improve its survival rate within two generations after heat shock selection experiments, indicating that the rapid increase in viability represents an important strategy for surviving harsh environments. These results illustrated that MED possessed a powerful regulatory plasticity system, along with a number of advantages over mammalian models including ethical acceptability, short generation times and the potential to investigate complex interacting parameters such as fecundity, longevity, gender ratio, and resistance to environmental stress, rendering it a suitable model for studying epigenetic adaptions. Therefore, we speculated that such short-term response mechanisms by MED to environmental temperature variations might be associated with DNA methylation. Although DNA methylation mechanisms have often been postulated as being involved in the rapid acquisition of adaptation trait and temperature resistance, the experimental evidence for this notion is scarce.

We considered several scenarios to determine the function of the *Dnmt1* gene under temperature stress conditions. First, we cloned the full MED *BtDnmt1* gene cDNA sequence and analysed the characteristics of the gene. Second, *BtDnmt1* dsRNA was fed to the whitefly, and *BtDnmt1* mRNA expression was examined using quantitative real-time PCR. Third, we identified the function of the *BtDnmt1* homology under knockdown and chill-coma temperatures by examining its survival rate biological statistics^55–57^. These data allow us to understand the thermal biology of the MED species. Furthermore, the data provided positive evidence that can explain phenotypic variation, thus revealing other invasive mechanism by which whitefly rapidly adapts to new environments.

## Results

### Sequence and characterization of *BtDnmt1*

**Figure 1:**
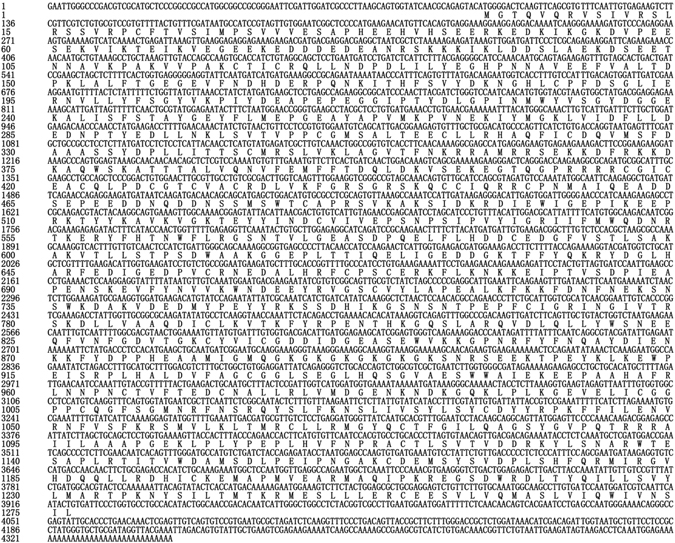
Nucleotide and deduced amino acid sequences of the *BtDnmt1* cDNA from MED.

The full-length cDNA of MED *BtDnmt1* is 5,958 bp and contains a 72-bp 5′-untranslated region (5′-UTR) (positions 1–72), a 1,599-bp 3′-UTR (positions 4360–5958) with a poly (A) tail and four tailing signals (Fig. 1), and a 4,287-bp open reading frame (positions 119–4362) that encodes a 1,428-amino-acid polypeptide with an estimated molecular mass of 160.9 kDa and an isoelectric point (pI) of 5.96.

**Figure 2:**
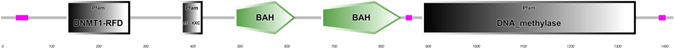
Conserved domains in MED BtDnmt1 analysed by SMART.

**Figure 3:**
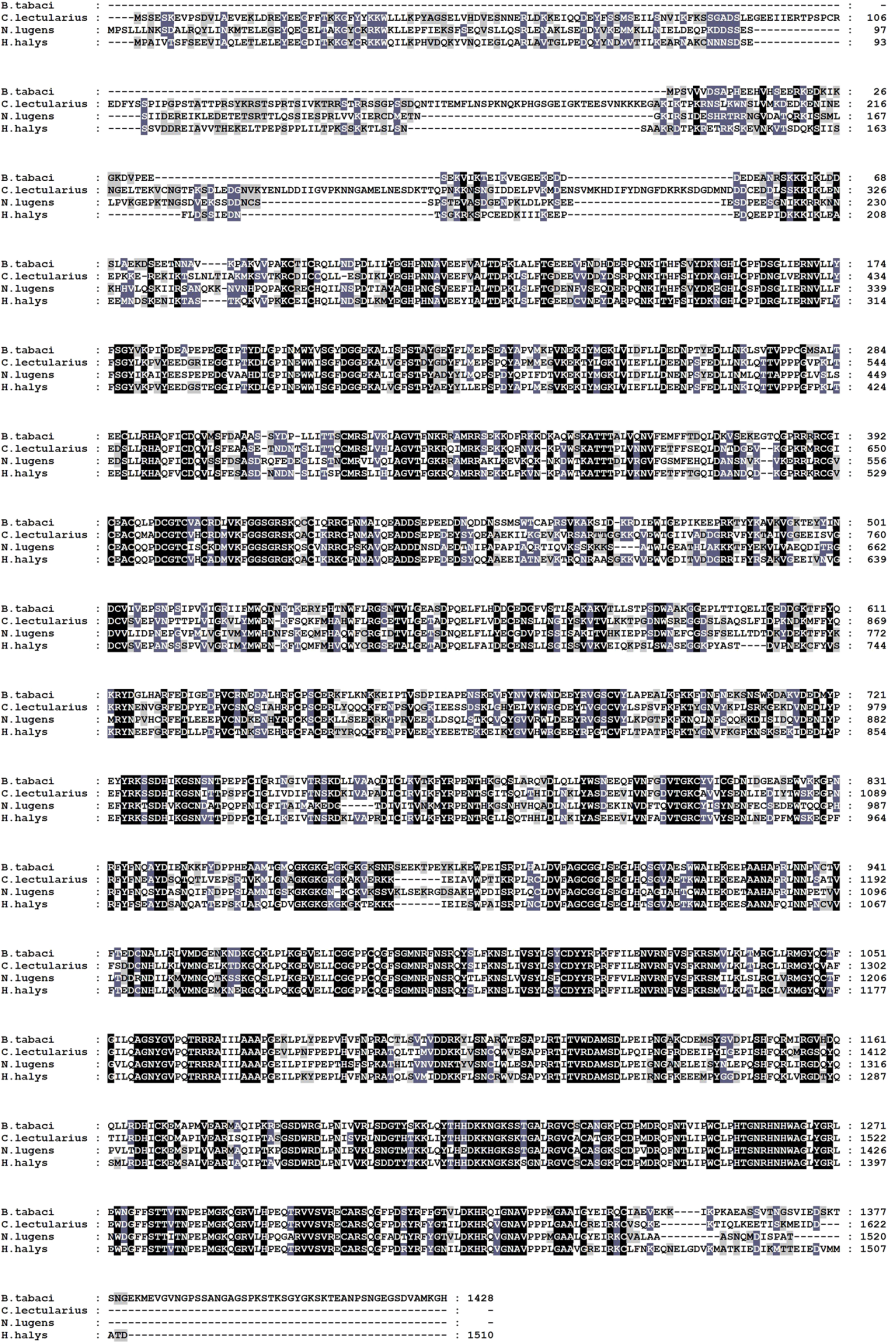
Multiple alignment of Dnmt1 proteins from *B. tabaci* MED and its homologues in the Hemiptera species. The deduced amino acid sequence of BtDnmt1 is highly conserved at catalytic domain when compared with previously identified Dnmt1 amino acid sequences, including *C. lectularius* (XP_014253428), *H. halys* (XP_014281484) and *N. lugens* (AHZ08393).

SMART software analysis (Fig. 2) also indicated that the deduced amino acid sequence of the BtDnmt1 protein is homologous to Dnmt1 that had typical structural features and contains a C-terminal catalytic domain (residues 894–1348) and an N-terminal regulatory domain (residues 139–850). The conserved domains characteristic of Dnmt1 showed a distinct multidomain structure harbouring a replication foci-targeting sequence (RFT) (residues 139–274), a zinc-finger-like (CXXC) motif (residues 382–428), and two tandemly connected bromo-associated homology (BAH) domains (residues 496–625, 680–850). The catalytic domain consists of six highly conserved motifs (I–X) (residues 894–1348). Furthermore, multiple sequence alignment of Dnmt1 proteins from different species revealed that BtDnmt1 was highly conserved especially in the C-terminal catalytic domain (Fig. 3), so it was a putative Dnmt1.

**Figure 4:**
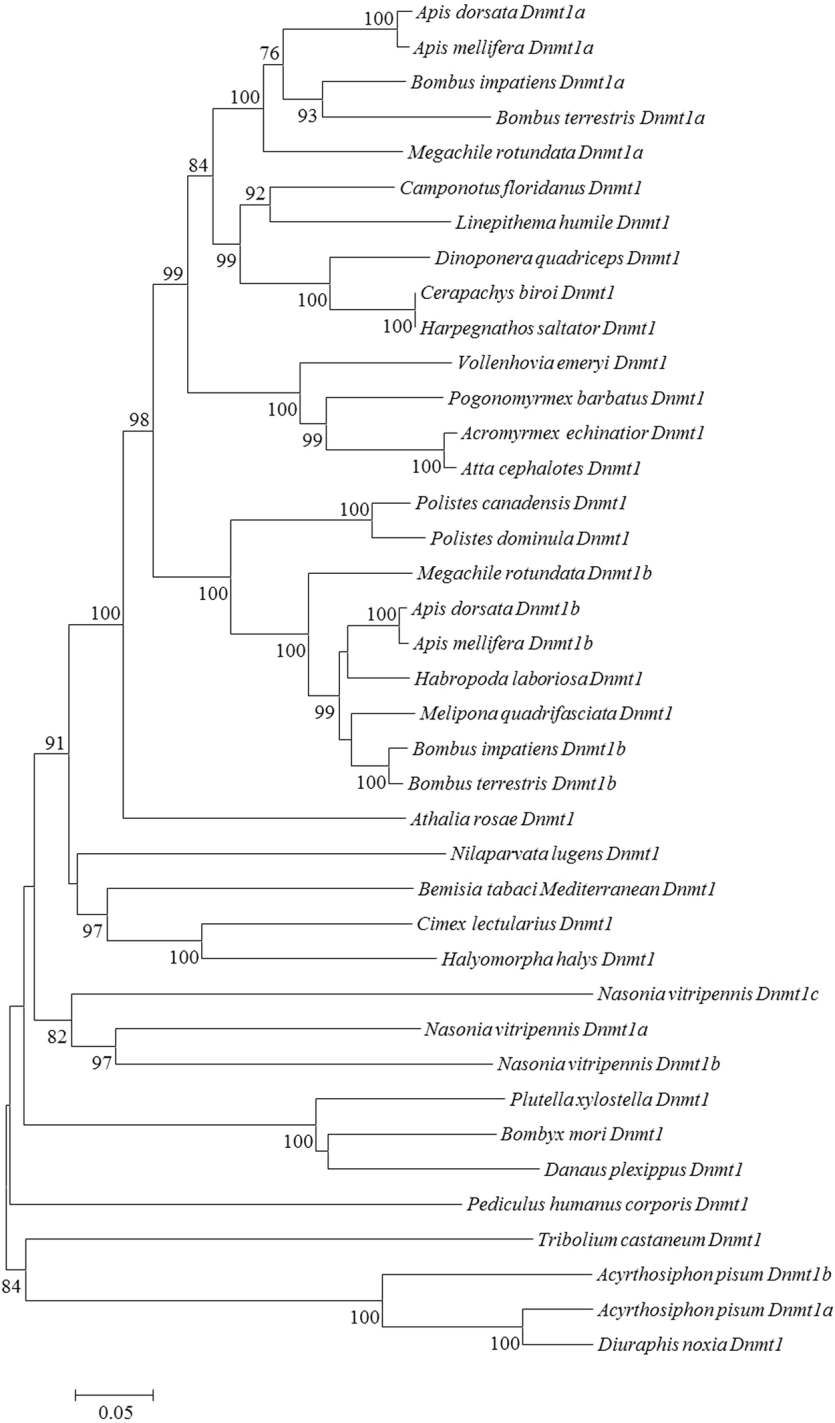
Phylogenetic relationships between Dnmt1 proteins from informative species. The phylogenetic tree was constructed with the maximum likelihood method using the MEGA 5 software. Bootstrap majority consensus values for 1,000 replicates are indicated at each branch point (%). The scale bar represents the branch length, which indicates an evolutionary distance of amino acid substitutions per position.

To examine the phylogenetic relationship between BtDnmt1 and other insects, a phylogenetic tree was constructed based on the deduced amino acid sequences from 31 species, which included five orders and 15 families. As shown in Fig. 4, these methyltransferases were grouped into most phylogenies with posterior probability values between 35 and 100. MED BtDnmt1 was most closely related to *Cimex lectularius* and *Halyomorpha halys* and clustered with a clade containing *Nilaparvata lugens* Dnmt1.

### *BtDnmt1* mRNA expression in *B. tabaci* after dsRNA feeding

**Figure 5:**
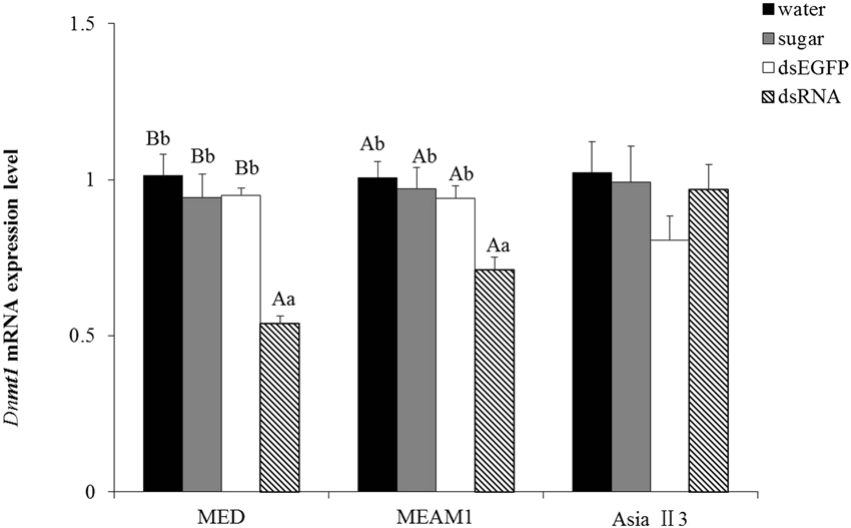
Effect of dsRNA treatment on *Dnmt1* mRNA expression in *B. tabaci*. *BtDnmt1* mRNA expression was significantly decreased in MED and MEAM1 after feeding with dsRNA for 3 h compared with expression in the controls. The results are expressed as the mean ± the SEM. The means with different lowercase letters above the bars are significantly different at P < 0.05. The means with different uppercase letters above the bars are significantly different at P < 0.01.

The primer sequences that were used to detect the relative amounts of *BtDnmt1* mRNA expression by real-time PCR are listed in Table 1. Melting curve analysis indicated that the primers used were specific for the *BtDnmt1* gene (Supplementary Figure 1). The amplification efficiency and standard curve of the *BtDnmt1*and *β-actin* primers were Y = −3.22× + 39.428 (R^2^ = 0.994) and Y = −3.326× + 48.642 (R^2^ = 0.991) (Supplementary Figure 2), respectively. Injection of dsRNA targeting the *EGFP* gene as an unrelated control gene did not affect the expression of the *BtDnmt1* gene studied in the three whiteflies indicating that the injection by itself did not interfere with gene expression. *BtDnmt1* mRNA expression was significantly decreased in MED (F_3,20_ = 9.033) and MEAM1 (F_3,20_ = 4.936) compared to that in the control treatments after ds*Dnmt1* feeding, but no significant difference in AsiaII3 (F_3,20_ = 0.944) (Fig. 5).

**Table 1:** Primer sequences used for cDNA cloning, real-time quantitative PCR and dsRNA synthesis.

Gene	Primer sequence (5′-3′)	Fragment length (bp)
PCR	CACGTTTGGAATCCTACAAGC	355
	TTCTGGCCTCAACCATTGGA	
*BtDnmt1*–3′ Outer	CCATGACCAACAACTTCTGCGAGACCA	2373
*BtDnmt1*–3′ Inner	CTCCAATGGTTGAGGCCAGAATGGCTC	
*BtDnmt1*–5′-1 Outer	CTGGCCTCACCATTGGAGCCATTTCTTTGCAG	2600
*BtDnmt1*–5′-1 Inner	GGCTGATTCGGTCCATCGAGCATTTGAGAGGT	
*BtDnmt1*–5′-2 Outer	AGCATCTTCATTCCGGCAGACAGGATCTTCAC	1544
*BtDnmt1*–5′-2 Inner	ACTGCCTTGTAGTACGTCTTGCGAGGCTCTTC	
**Real-time quantitative PCR**		
*BtDnmt1*-F	ATCGTGTCGGCAGTTGCGTCTA	181
*BtDnmt1*-R	AGGGTTCTGGCGTGTTGGAG	
*β-actin*-F	TCACCACCACAGCTGAGAGA	231
*β-actin*-R	CTCGTGGATACCGCAAGATT	
**dsRNA synthesis primers**		
ds*BtDnmt1*-F	TAATACGACTCACTATAGGGAGACCACATCGTGTCGGCAGTTGCGTCTA	258
ds*BtDnmt1*-R	AGGGTTCTGGCGTGTTGGAG	
ds*EGFP*-F	TAATACGACTCACTATAGGGTGAGCAAGGGCGAGGAG	678
ds*EGFP*-R	TAATACGACTCACTATAGGGCGGCGGTCACGAACTCCAG	

Primer sequences plus T7 promoter sequences (underlined) are shown for production of the dsRNA transcription templates.

### Survival rate under temperature stress conditions after dsRNA feeding

**Figure 6:**
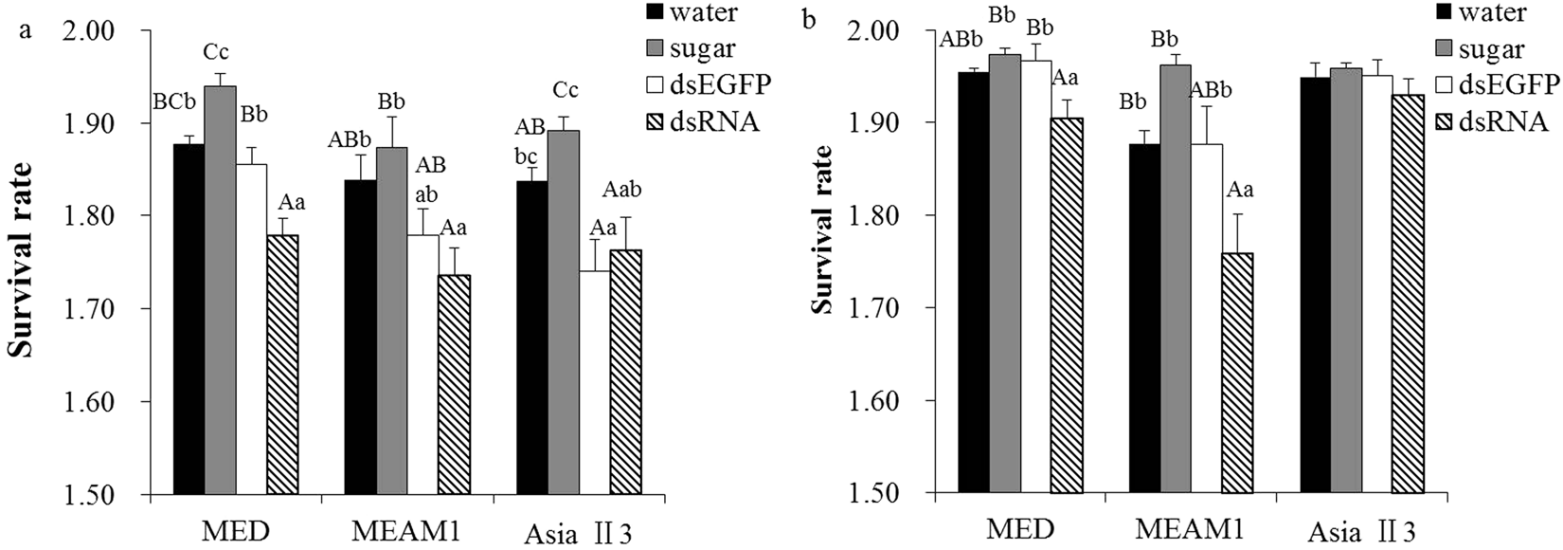
Effect of *Dnmt1* dsRNA treatment on the temperature-resistance of *B. tabaci* adults. Feeding with *BtDnmt1* dsRNA significantly decreased survival rates at (**a**) 45 °C and (**b**) −5 °C in two invasive whiteflies, MED and MEAM, compared with the survival rates in the control treatments, suggesting that Bt*Dnmt1* is a key factor influencing the temperature tolerance of invasive species. The results are expressed as the mean ± SEM. The means with different lowercase letters above the bars are significantly different at P < 0.05. The means with different uppercase letters above the bars are significantly different at P < 0.01.

As shown in Fig. 6a, compared with the control treatments, the survival rate was extremely significantly decreased at 45 °C for 1 h after feeding with BtDnmt1 dsRNA in MED (F_3,20_ = 25.834, P < 0.01); and the survival rate in MEAM1 was also significantly decreased compare to water and sugar fed control (F_3,20_ = 5.616, P < 0.05). However, there was only a significant difference between feeding ds*Dnmt1* and sugar in AsiaII3 (F_3,20_ = 8.392, P < 0.01). The survival rates of feeding ds*Dnmt1*, ds*EGFP*, sugar and water in MED were 60.4%, 71.7%, 87.1%, and 75.4%, respectively; the rates in MEAM1 were 55.1%, 60.3%, 74.7% and 70.0%, respectively; and the rates in AsiaII3 were 58.9%, 55.6%, 78.1% and 63.2%, respectively.

Furthermore, as shown in Fig. 6b, compared with the control treatments, the survival rates were significantly decreased at −5 °C for 1 h after feeding with *BtDnmt1* dsRNA both in MED (F_3,20_ = 8.701, P < 0.05) and MEAM1 (F_3,20_ = 13.287, P < 0.05), and no significant difference was found in AsiaII3. The survival rates of feeding ds*Dnmt1*, ds*EGFP*, sugar and water in MED were 60.4%, 92.6%, 87.1%, and 75.4%, respectively; the rates in MEAM1 were 55.1%, 75.6%, 74.7% and 70.0%, respectively; and the rates in AsiaII3 were 85.6%, 89.5%, 91.0% and 88.2%, respectively. The results showed that *BtDnmt1* influenced temperature tolerances in MED and MEAM1, and there were differences in heat tolerances of MED and MEAM1.

## Discussion

The present results demonstrate that BtDnmt1 is a putative DNA methyltransferase 1 that contains all the characteristic domains and motifs for maintaining DNA methyltransferase activity (Fig. 1). The N-terminal domain had multiple regulatory mechanisms that controlled the activity and specificity of DNA methylation^58, 59^. The N-terminal domain also contained a nuclear localization signal (NLS) and a RFT domain that localized Dnmt1 to the DNA replication fork^60^, a cysteine-rich zinc finger domain that specifically recognized unmethylated CpG DNA^61^, and two BAH domains that targeted Dnmt1 to replication foci^62^. N-terminal sequences from different species might coordinate with different chromatin structures with large variations, but the important domains, including RFT, CXXC, and BAH, were highly conserved. The C-terminal catalytic domain harbours 6 highly conserved motifs that maintain essential Dnmt1 CpG methylation patterns through successive DNA replication rounds, thereby preventing cell death^63^. Furthermore, a phylogenetic tree was constructed based on the highly conserved catalytic domains of these proteins, and Dnmt1s from insects from the same order were clustered into the same group, consistent with traditional taxonomy. Moreover, the C-terminal sequence of MED exhibited 76% similarity with *N. lugens* and more than 60% similarity with other insects, suggesting that the role of the Dnmt1 is relatively conserved across species^64^.

Studies have shown that the mammalian Dnmt1 family includes Dnmt1s, Dnmt1o and Dnmt1p^22, 64^. Dnmt1s was expressed in somatic cells, Dnmt1o was specific to oocytes and preimplantation embryos, and Dnmt1p was found only in pachytene spermatocytes^64^. Dnmt1o (Genbank accession number: NP_001186362.1) has a shorter 118-amino-acid Dnmt1-associated protein 1(DMAP1) binding region (UniProt accession number: P13864) than Dnmt1s^65–67^. Intriguingly, the DMAP1 interaction domain was not present in the N-terminal domains when using either CDD or SMART to deduce the MED BtDnmt1 structure, a finding that is consistent with *N. lugens* Dnmt1. Therefore, we speculated that MED *BtDnmt1* belonged to an autologous gene of mammalian *Dnmt1o*. DMAP1 was initially identified as a protein that was associated with the N-terminal domain of Dnmt1, which co-localizes with PCNA at the DNA replication foci during the S phase and is associated with Dnmt1 maintenance^66^. DMAP1 was subsequently demonstrated to be a component of the histone acetyltransferase complex^68, 69^. In mammals, DMAP1 plays a crucial role in DNA repair, acts against genomic instability, is indispensable for maintaining chromosomal integrity^70, 71^, and might be able to control Dnmt1s degradation. Dnmt1o was eventually excluded from DMAP1 regulation and consequently became a more stable protein than Dnmt1s^72^. In mice, DMAP1-Dnmt1s and DMAP1-Dnmt1o interactions are essential for normal development, although DMAP1-Dnmt1o complexes do not readily form in the embryo. Considering the above findings, the whitefly samples collected included all ages with six replicates. All the results suggested that the Dnmt1 in MED is a homolog of Dnmt1o, which is more stable than Dnmt1s and has a correspondingly high enzymatic activity, which is consistent with previous studies. Thus, it was concluded that the putative BtDnmt1o is essential for regulating methylation patterns to ensure a rapid response to environmental temperature variations, which was important for the underlying invasion process by MED.

Invasive MED can adapt to various climate regions and might possess a unique thermal adaptability mechanism. Previous studies have demonstrated that DNA methylation, as an important environment-induced mechanism, can serve as a potential link between phenotypic variability and temperature variation, which is reprogrammed by Dnmts. For instance, Campos *et al*.^73^ reported that increasing embryonic temperature ranges in fish had remarkable dynamic effects on *Dnmt3* gene expression. Furthermore, Yan *et al*.^74^ showed that *Dnmt1* might be involved in the thermal epigenetic regulation of embryos during early development in ducks. Based on these data, we used the RNAi method to identify the function of the Bt*Dnmt1* gene under high and low temperature conditions. The results showed that feeding with *BtDnmt1* dsRNA significantly decreased survival rates in two invasive whiteflies, MED and MEAM1, thus demonstrating that *BtDnmt1* homology plays an essential role in the thermotolerance of the invasive species. Furthermore, the survival rate after exposure to 45 °C was significantly higher in MED than in MEAM1, indicating that MED is more tolerant to high temperatures than MEAM1. This observation has important implication for understanding the geographic distribution and displacement of the two invasive species in the field. However, additional studies using methods such as bisulphite sequencing are required to confirm the potential links between temperature and epigenetic modification, to investigate the underlying molecular mechanisms, and to identify the precise targets and levels of DNA methylation under different temperature stresses.

## Conclusions

To our knowledge, this study is the first to reveal the characteristics of *BtDnmt1* in invasive MED and to identify the functions of *BtDnmt1* homology using RNAi. The results indicated that BtDnmt1 homology plays an important role in the thermotolerance of invasive species *B. tabaci* and provide a new direction for studying the rapid adaptability of invasive *B. tabaci*. These findings should aid in further interpreting the population expansion and invasion mechanisms of this invasive species.

## Materials and Methods

### Insect and plant samples

Three whitefly species, MED, MEAM1 and AsiaII3, were maintained, without laboratory exposure to insecticides, on tomato plants (*Lycopersicon esculentum* Mill (Zhongza No. 9)) in cages in an insectary at 24–32 °C under 50–60% relative humidity with a 14:10 h light:dark cycle. The plants were individually grown in 9-cm-diameter pots under the same conditions as the whitefly.

### RNA extraction and cDNA synthesis

Total RNA was extracted from approximately 200 MED adults using TRIzol reagent (Invitrogen, Carlsbad, CA, USA) following the manufacturer’s protocol. RNA was quantified using a NanoPhotometer^TM^ P330 instrument (Implen, Munich, Germany), and the A260/A280 ratio was typically above 2.0. The RNA quality was also evaluated via 1% agarose gel electrophoresis. Reverse transcription was performed using 2.0 µg of each RNA sample in a 20.0 µL reaction with an oligo(dT)_18_ primer according to the instructions provided with the Super Script First-Strand Synthesis System (Transgen, Beijing, China).

### Full-length cDNA cloning of the *BtDnmt1* gene

Primers were designed based on the transcriptome information of *B. tabaci*, and were used to amplify partial segments of the Bt*Dnmt1* gene. Next, rapid amplification of cDNA ends (5′- and 3′-RACE) was performed to obtain the full-length cDNAs using a SMART RACE cDNA amplification kit (Clontech, Mountain View, CA, USA) according to the manufacturer’s instructions. The gene-specific primer sets (Table 1) were designed based on the *B. tabaci* transcriptome information from the NCBI website (http://www.ncbi.nlm.nih.Gov). The amplified fragments were purified using an AxyPrep^TM^ DNA Gel Extraction Kit (Axygen, West Orange, NJ, USA). Finally, the distinct single-band amplification products were cloned into the pEASY-T3 vector (Transgen, Beijing, China) and sequenced.

### Bioinformatics characterization of the *Dnmt1* gene

Sequence alignment and identity analyses were performed using DNAMAN (version 5.0; LynnonBioSoft, Quebec, Canada). Open reading frames (ORFs) were identified using ORF Finder (http://www.ncbi.nlm.nih.gov/gorf/orfig.cgi). Molecular weights and pIs were calculated using ExPASy (http://web.expasy.org/protparam/). Conserved functional domains of the deduced *BtDnmt1* protein sequence were identified using SMART software (http://smart.embl-heidelberg.de/). Multiple protein sequences were aligned using Clustal W as implemented in the MAGE 5.2.2 software package to evaluate the molecular evolutionary relationship between *BtDnmt1* genes from various insects^75^. The phylogenetic tree was constructed using the Neighbour-Joining method in MAGE 5.2.2 with a bootstrap value of 1,000.

### Production of the dsRNA transcription templates and dsRNA synthesis

*BtDnmt1* transcription templates were produced from total *B. tabaci* cDNA using specific primers that were conjugated using a T7 RNA polymerase promoter (Table 1). Template amplification reactions contained 2.0 µg of cDNA template, 2.5 U of TransStartTaq DNA Polymerase, 5.0 µL of 10× buffer, 200 µM of each dNTP, 400 µM of forward primer, and 400 µM of reverse primer in a total volume of 50.0 µ. The following PCR cycling conditions were used for amplification: (1) 94 °C for 5 min, followed by 35 cycles of 94 °C for 30 s, 62 °C for 30 s, and 72 °C for 30 s, and (3) a final extension step of 72 °C for 10 min. The amplified PCR products were resolved on and purified from 1.0% agarose gels as described above.

*In vitro* double-stranded RNA (dsRNA) synthesis was performed using the MEGAscriptT7 High Yield Transcription Kit (Ambion, Austin, USA). The transcription reaction contained 1 µg of purified products as the transcription template, 7.5 mM of each ribonucleotide, and 200 U of T7 enzyme mix in the appropriate buffer in a final volume of 20.0 μL.

The reactions were incubated at 37 °C for 6 h; then 2 U of TURBO DNase was added, and the mixture was incubated at 37 °C for 15 min. Then, 7.5 M LiCl precipitation solution (30 μL) was added to the reaction to purify the dsRNA at −20 °C for 30 min, followed by centrifugation at 15,000 rpm for 15 min at 4 °C. Finally, the RNA pellet was washed with 70% ethanol and resuspended in diethyl pyrocarbonate (DEPC)-treated water. dsRNA quality and concentration were determined by 1.0% agarose gel electrophoresis and NanoPhotometer spectrophotometer measurement. The dsRNA was then stored at −80 °C until use.

### dsRNA feeding and detection

Newly emerged MED, MEAM1 and AsiaII3 adults were fed a diet containing dsRNA diluted to 0.3–0.5 μg/μL in a 10% w/v RNase-free sucrose solution. Feeding was performed using the Parafilm clip nutrient solution method^76, 77^. The Parafilm was pre-treated with 0.1% (DEPC solution to remove any RNase, and RNase-free water was used to clean DEPC from the Parafilm. Two hundred newly emerged whitefly adults were collected and placed in a glass tube (3 cm in diameter ×8 cm in height)). The tube opening was then covered with two layers of Parafilm, and 200–250 μL of dsRNA solution was injected into the gap between the layers. The other end of the tube was covered with gauze to enable ventilation. The tube was then wrapped with black plastic paper, leaving the Parafilm-enclosed end exposed to light. This process encouraged the adults to move towards the diet and feed. Each tube was then placed in an artificial climate box (Safe, Ningbo, China) at 26 ± 0.2 °C for 3 h. At 3 h, some of the samples were immediately frozen in liquid nitrogen and were stored at −80 °C until RNA extraction. The remaining whiteflies were exposed to temperatures of 45 ± 0.2 °C or −5 ± 0.2 °C in a water bath for 1 h, after which they were then placed into another constant environment room at 26 ± 0.2 °C for 1 h; the number of live whiteflies was then counted. The samples at 26 °C were used as a control. The temperatures of 45 °C and −5 °C were selected based on preliminary experiments showing that these temperatures were discrimination points for whitefly temperature tolerance. Treated controls were fed with 10% w/v RNase-free sucrose solution or with an enhanced green fluorescent protein (EGFP)-specific dsRNA, and the untreated control was fed water. Each treatment had six replicates.

Total RNA from the samples that had fed dsRNA on was extracted using the TRIzol reagent. RNA quantity and quality of the RNA were assessed using A260/A280 ratio that was measured using a NanoPhotometer^TM^ P330, which were typically above 2.0. The RNA quality was also evaluated via 1% agarose gel electrophoresis. Two micrograms of total RNA was used to synthesize cDNAs using the Super Script First-Strand Synthesis System according to the manufacturer’s instructions. The cDNA was stored at −80 °C until further analysis.

*BtDnmt1* mRNA expression after feeding with dsRNA was evaluated by quantitative real-time PCR analysis. The primer sequences used are listed in Table 1. The reactions were performed using an ABI 7500 Real-time PCR system (Applied Biosystems, USA). All amplifications were confirmed by sequencing, and the specificity of qRT-PCR reactions was estimated by melting curve analysis. PCR assays were prepared with 1.0 µL of the cDNA template, 10.0 µL of 2× TransStart^TM^ Green qPCR SuperMix, 200 µM of each gene-specific primer (Table 1), and 0.4 µL of Passive Reference Dye in a final volume of 20.0 μL. A thermocycler was programmed with the following cycling conditions: (1) 94 °C for 1 min, followed by (2) 40 cycles of 95 °C for 15 s, 61 °C for 30 s and 72 °C for 30 s. Each sample was assessed in triplicate (technical replicates). A control without the cDNA template was included in all batches. *β-actin* was used as the reference gene because it is constitutively expressed under various temperature stress conditions^78^. Amplification efficiency was validated by constructing a standard curve using five serial dilutions of cDNA. The relative quantification of *BtDnmt1* mRNA expression was calculated using the mathematical model of Livak & Schmittgen^79^, which simplifies to 2^−ΔΔCT^ as follows: (ΔΔCT = (Ct_target_ − Ct_reference_)_treatment_ − (Ct_target_ − Ct_reference_)_control_). The relative *BtDnmt1* mRNA expression level was defined as the fold change compared to the amount of *β-actin*. Each sample was assessed in triplicate.

### Statistical analysis

Statistical analyses were performed using the SPSS v. 16.0 software package (SPSS Inc., Chicago, IL, USA). Data were first tested for normality using the Kolmogorov-Smirnov test. All percentage data were log-transformed to ensure that they were normally distributed. Target gene mRNA expression and survival rate after feeding with the dsRNA mixture were analysed using one-way ANOVA followed by Fisher’s least significant difference (LSD) test. The data were presented as means ± standard errors (mean ± SE). Differences were considered statistically significant when P ≤ 0.05.

## Electronic supplementary material

Supplementary
