# Supplementary material for: The homology gene BtDnmt1 is Essential for Temperature Tolerance in Invasive Bemisia tabaci Mediterranean Cryptic Species
Source: Sci Rep. 2017 Jun 8;7:3040. doi: 10.1038/s41598-017-03373-w (PMC5465089; doi:10.1038/s41598-017-03373-w)
Supplement: Supplementary file 1 — Supplementary [file 41598_2017_3373_MOESM1_ESM.doc]

**Article Title:** The homology gene *BtDnmt1* is Essential for Temperature Tolerance in Invasive *Bemisia tabaci* Mediterranean Cryptic Species

**Author list:**  Tian Mei Dai, Zhi Chuang Lü, Wan Xue Liu, Fang Hao Wan, Xiao Yue Hong


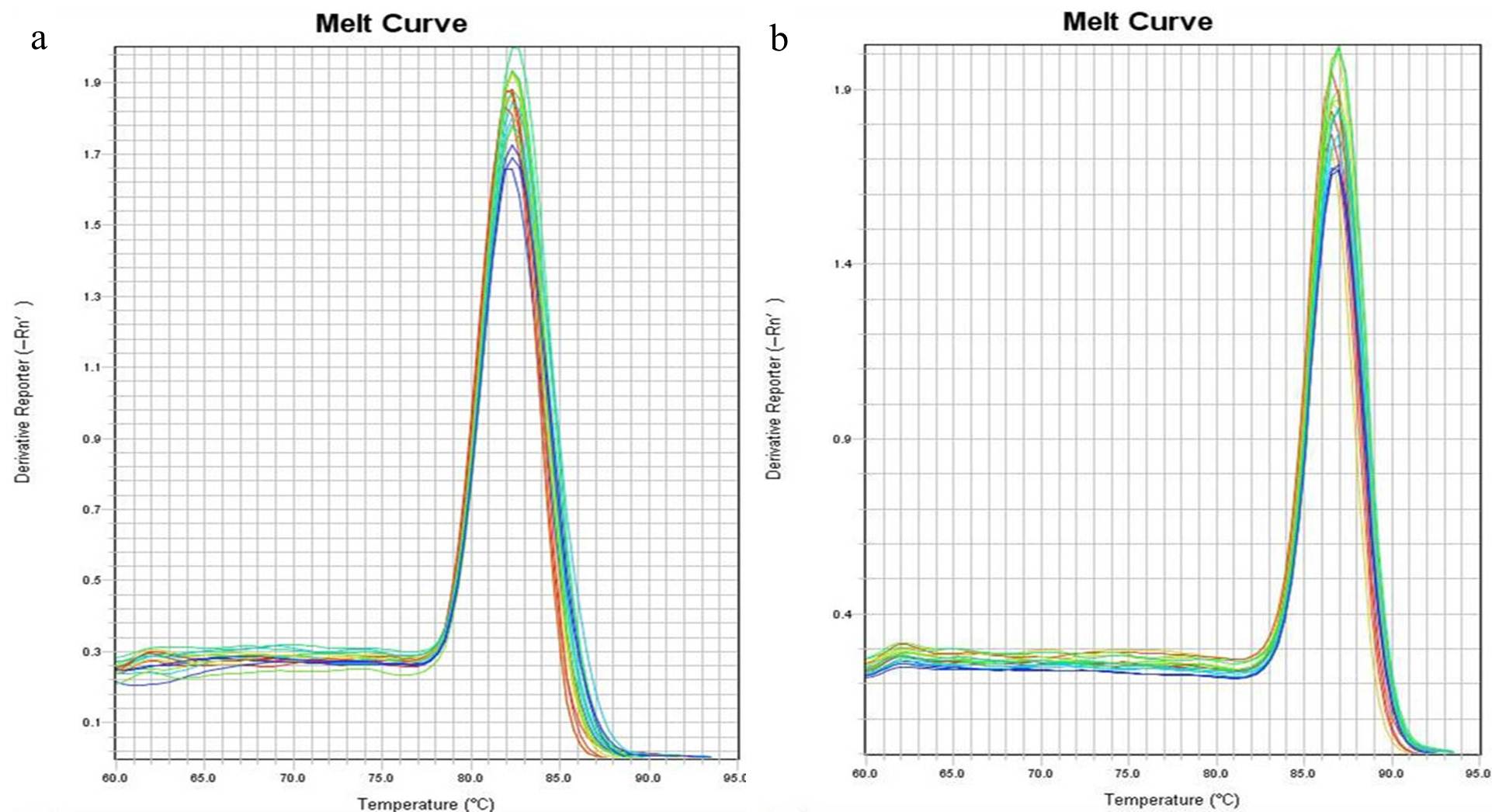


Supplementary figure 1. Dissociation Curve

a: *BtDnmt1* ; b: *β-actin*

*
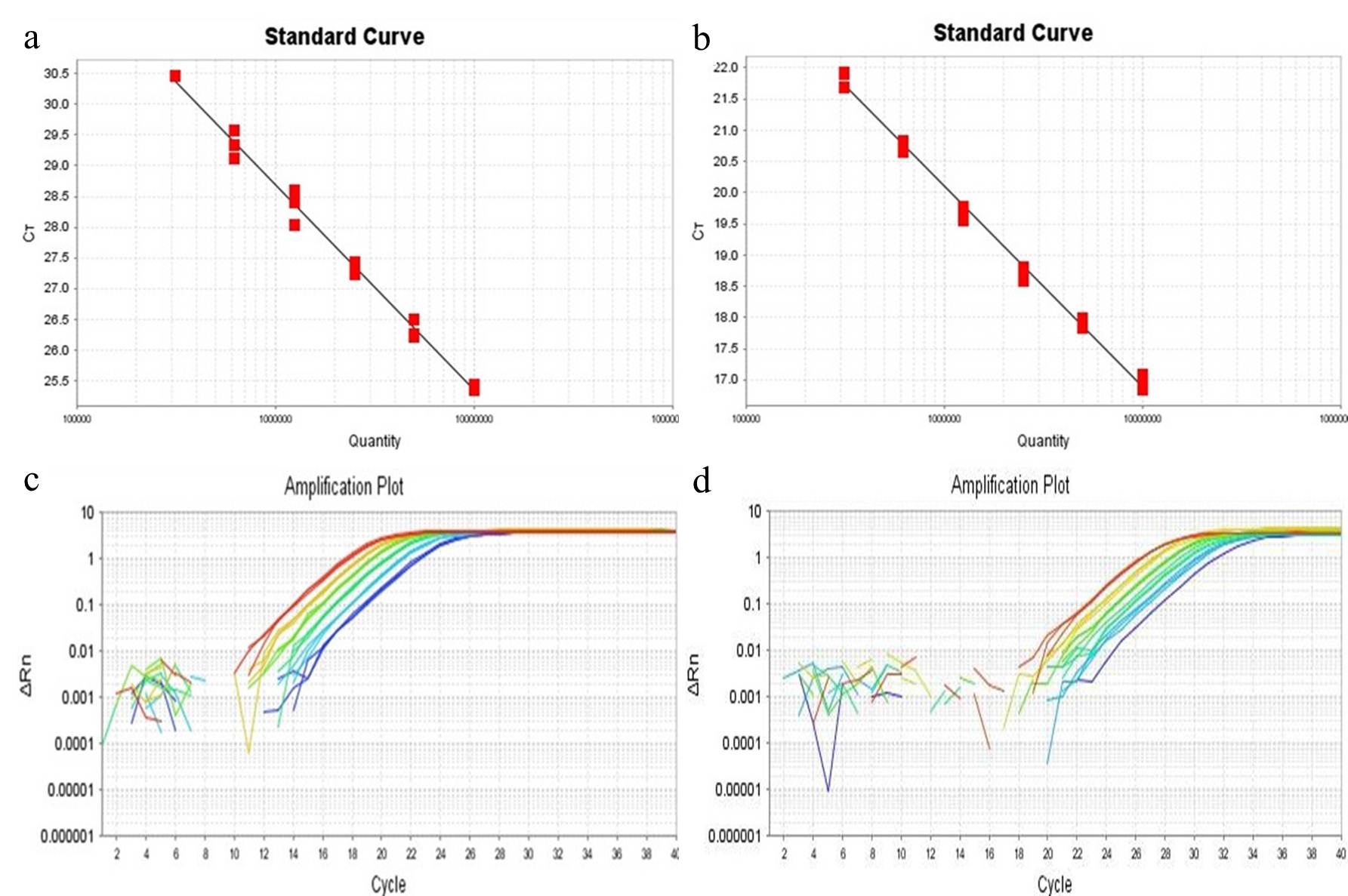
*

Supplementary figure 2. Standard curve and amplification plot from selected differentially expressed genes

a: standard curve of *BtDnmt1* ; b: standard curve of *β-actin*; c: amplification plot of *BtDnmt1* ; d: amplification plot of *β-actin*
